# Pyodermatitis-pyostomatitis vegetans: a case report and systematic review focusing on oral involvement

**DOI:** 10.1007/s10006-024-01234-1

**Published:** 2024-03-12

**Authors:** Bruno Ramos Chrcanovic, Roberta Rayra Martins-Chaves, Flávia Sirotheau Correa Pontes, Felipe Paiva Fonseca, Hélder Antônio Rebelo Pontes, Ricardo Santiago Gomez

**Affiliations:** 1 Department of Oral and Maxillofacial Surgery and Oral Medicine, Faculty of Odontology, Malmö University, Carl Gustafs väg 34, Malmö, SE-214 21 Sweden; 2 Department of Oral Pathology, João de Barros Barreto University Hospital, Universidade Federal do Pará, Belém, Brazil; 3 Faculdade de Ciências Médicas de Minas Gerais, Belo Horizonte, Brazil; 4 Department of Oral Surgery and Pathology, School of Dentistry, Universidade Federal de Minas Gerais, Belo Horizonte, Brazil

**Keywords:** Pyodermatitis-pyostomatitis vegetans, Pyostomatitis vegetans, Inflammatory bowel disease, Systematic review

## Abstract

**Background:**

Pyodermatitis-pyostomatitis vegetans (PPV) is a rare mucocutaneous disease characterized by multiple pustules and it is considered a marker for inflammatory bowel disease (IBD). The oral manifestations of this condition are referred to as pyostomatitis vegetans (PSV).

**Purpose:**

To investigate which features could help in establishing the diagnosis of PSV, with or without cutaneous lesions, based on information retrieved from all cases of PSV described in the literature. A case of PV from the authors was also included in the analysis.

**Methods:**

An electronic search was undertaken, last updated in August 2022. Inclusion criteria included publications reporting cases of PSV, with the diagnosis confirmed by the pathological examination of oral or skin lesions, and presence of IBD.

**Results/Conclusions:**

Sixty-two publications with 77 cases of PSV and an associated IBD were included. Features that are helpful in establishing the diagnosis of PSV are snail track appearance of oral lesions, an associated IBD (which is not always symptomatic), evidence of intraepithelial clefting on microscopic examination of oral lesions, and peripheral blood eosinophilia. A gold standard for the management of PSV does not exist and high-level evidence is limited. There is no established therapeutic protocol for PSV and management primarily consists of topical and/or systemic corticosteroids, antirheumatic drugs (sulfasalazine, mesalazine), monoclonal antibody (infliximab, adalimumab) immunosuppressives (azathioprine, methotrexate), antibiotics (dapsone), or a combination of these. The risk of recurrence of oral lesions is considerable when the medication dose is decreased or fully interrupted.

**Supplementary Information:**

The online version contains supplementary material available at 10.1007/s10006-024-01234-1.

## Introduction

Pyodermatitis-pyostomatitis vegetans (PPV) is a rare mucocutaneous disease characterized by multiple pustules and it is considered a marker for inflammatory bowel disease (IBD) [1, 2]. The cutaneous manifestations of this condition are referred to as *pyodermatitis vegetans* (PDV), usually presenting as erythematous, vesiculopustular, exudative, vegetating plaques often localized in the inguinal and axillary folds. *Pyostomatitis vegetans* (PSV), in contrast, commonly presents as confluent oral pustules and erosions termed ‘snail track’ lesion [3, 4].

Since the first descriptions of the disease in the German medical literature at the end of the 19th century [5, 6] and in the English literature half a century later [4], oral cavity involvement has been widely reported in PPV-affected patients. Despite the high frequency of oral lesions and the possible occurrence of PSV alone, a recent systematic review on PPV has excluded cases without cutaneous lesions [7]. Therefore, we systematically reviewed the literature on PSV, together with the report of an additional PPV case.

## Case report

**Fig. 1:**
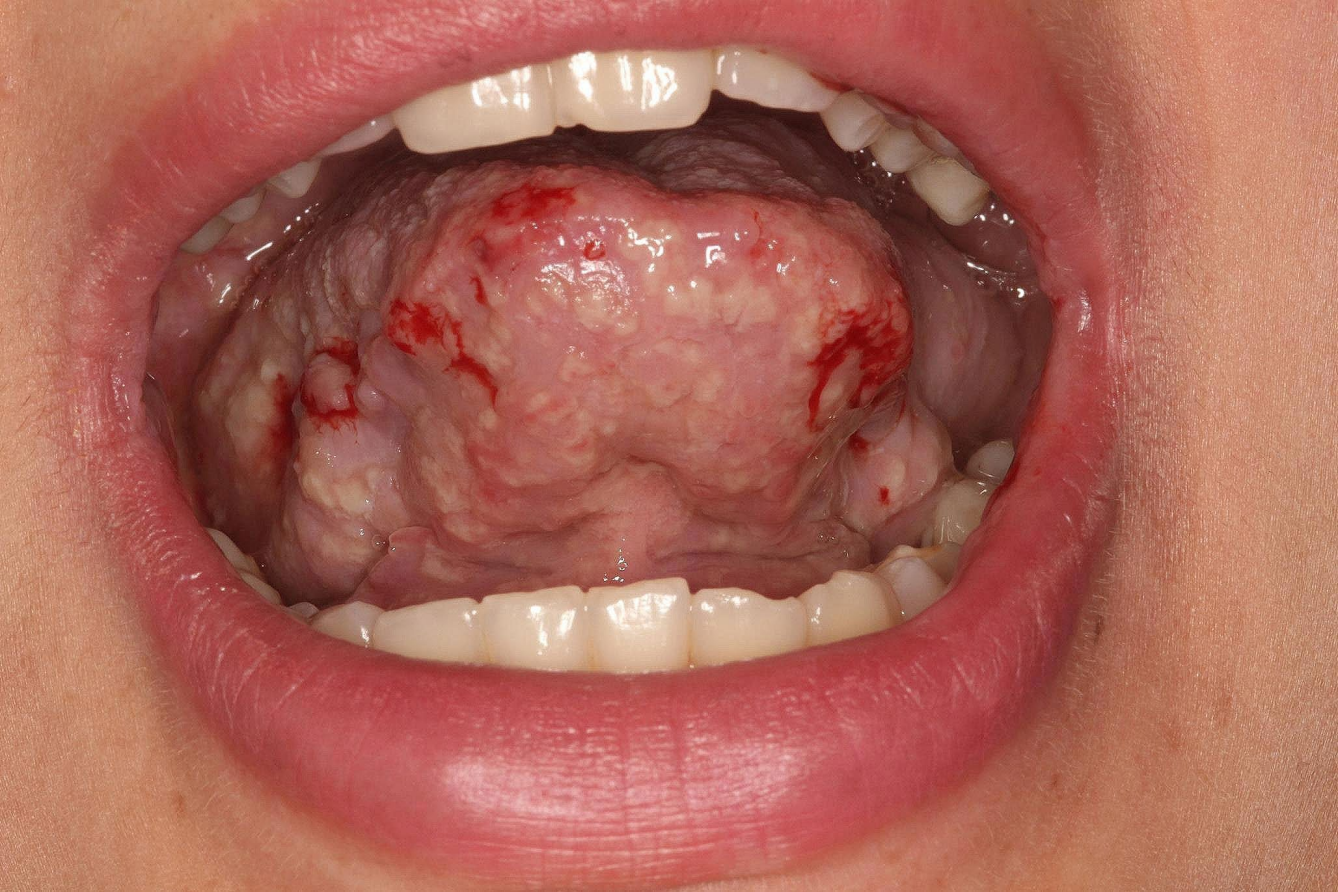
Clinical presentation of pyostomatitis vegetans affecting the ventral and lateral borders of the tongue at the initial visit. Notable are exophytic pustular lesions with a “snail track” appearance, erythematous background, and areas of hemorrhage

**Fig. 2:**
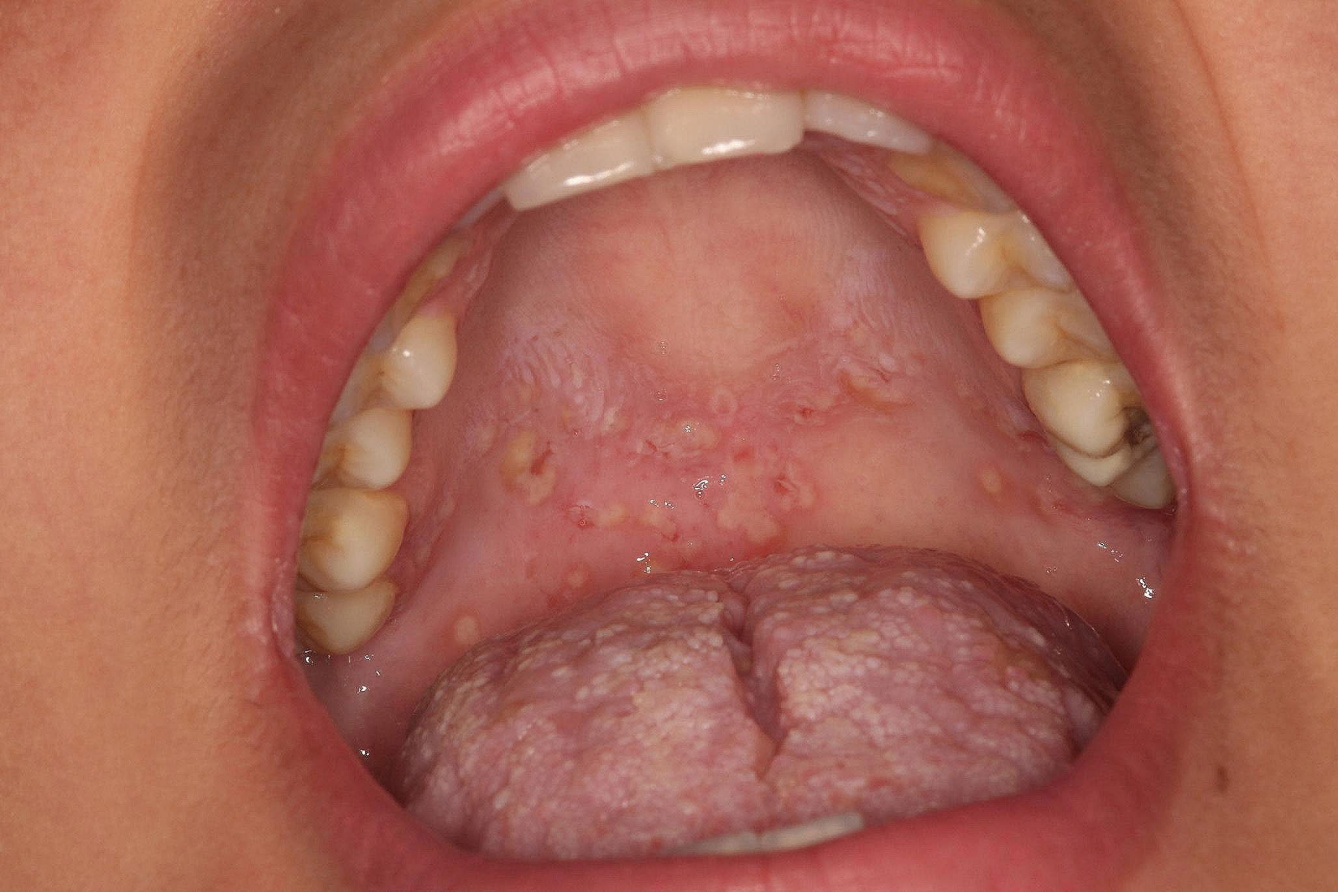
“Snail track” lesions involving palatal mucosa

**Fig. 3:**
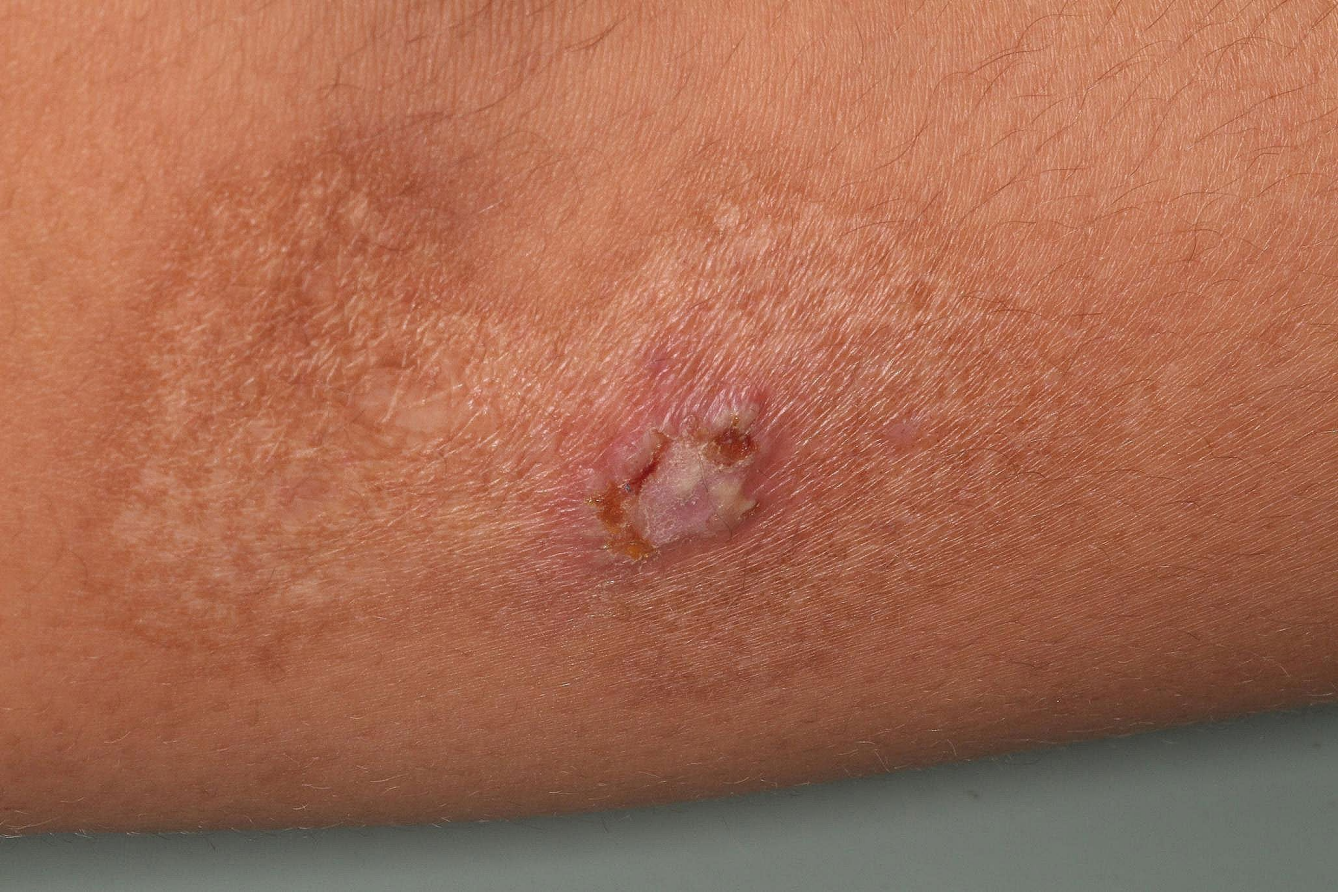
Pyodermatitis vegetans involving patient’s thigh and manifesting with yellowish crusts surrounded by erythema

**Fig. 4:**
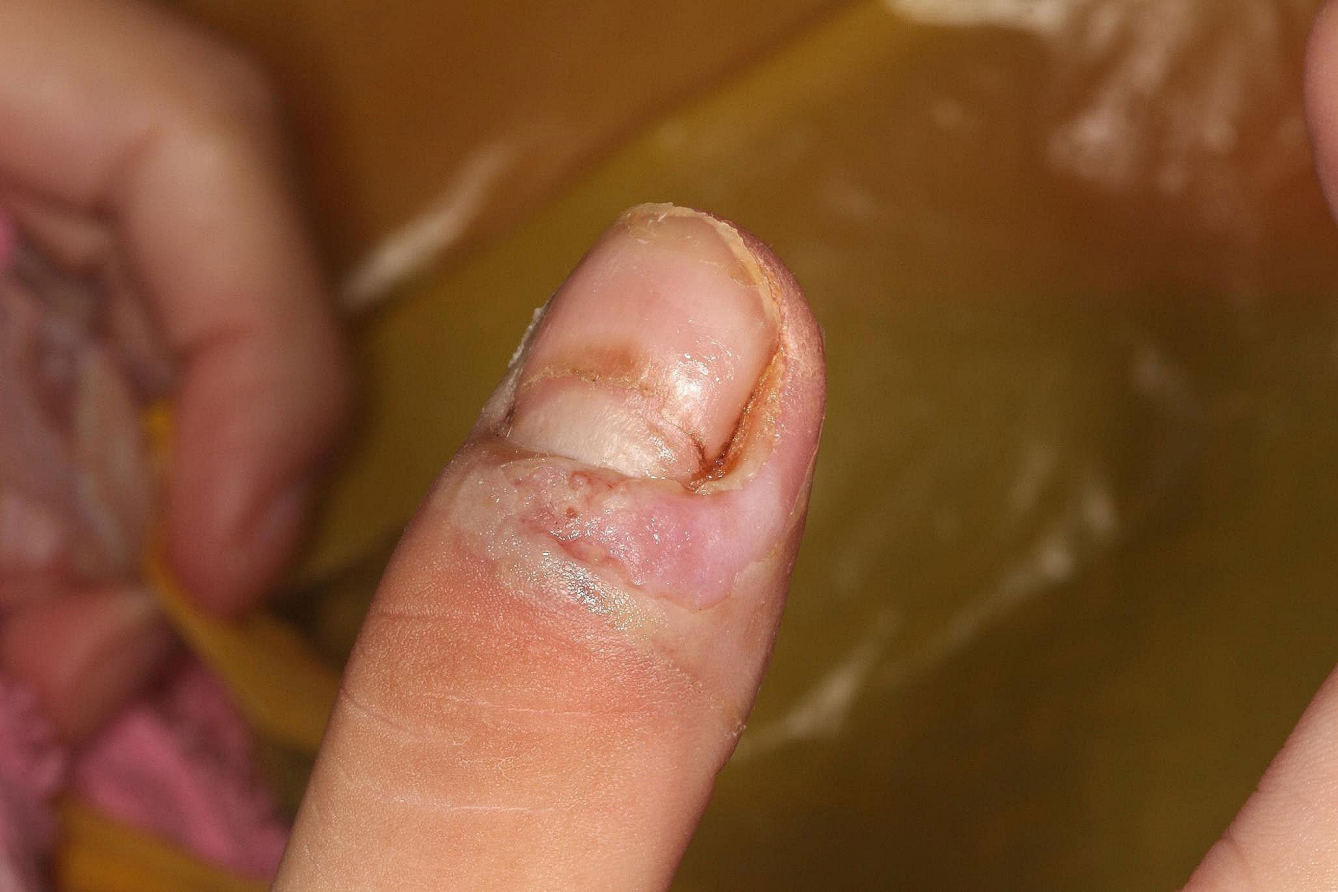
Pyodermatitis vegetans involving the patient’s finger at the initial visit

An 18-year-old female patient was referred to the Oral Diagnosis Service of the *João de Barros Barreto* University Hospital, Belém, Brazil, for evaluation of oral lesions of 2-month duration. The lesions were located on the floor of the mouth, buccal and labial mucosa, palate, upper and lower lip, gums and tongue. Lesions were exophytic with a yellowish background, in some areas appeared erosive, associated with bleeding (lip and posterior portion of the palate) and had a “snail track” appearance (Figs. 1 and 2). On the skin, yellowish crusts surrounded by reddened areas were observed in the groin and thigh (Fig. 3). Skin lesions were also observed in a hand finger (Fig. 4). Blood work indicated eosinophilia (10%, 834/mm^3^), and elevated erythrocyte sedimentation rate (50 mm/hr). The previous medical history was non-contributory. The patient was pregnant, with delivery scheduled for 20 days after the initial consultation. Despite reporting pain on eating, she refused to receive any treatment or further exam until after child birth. Amoxicillin with K clavulanate and dexamethasone mouthwash were prescribed for 10 days. Twenty days after birth, the patient returned to our Service, at which time an incisional biopsy of the lesion in the lower lip was performed.

**Fig. 5:**
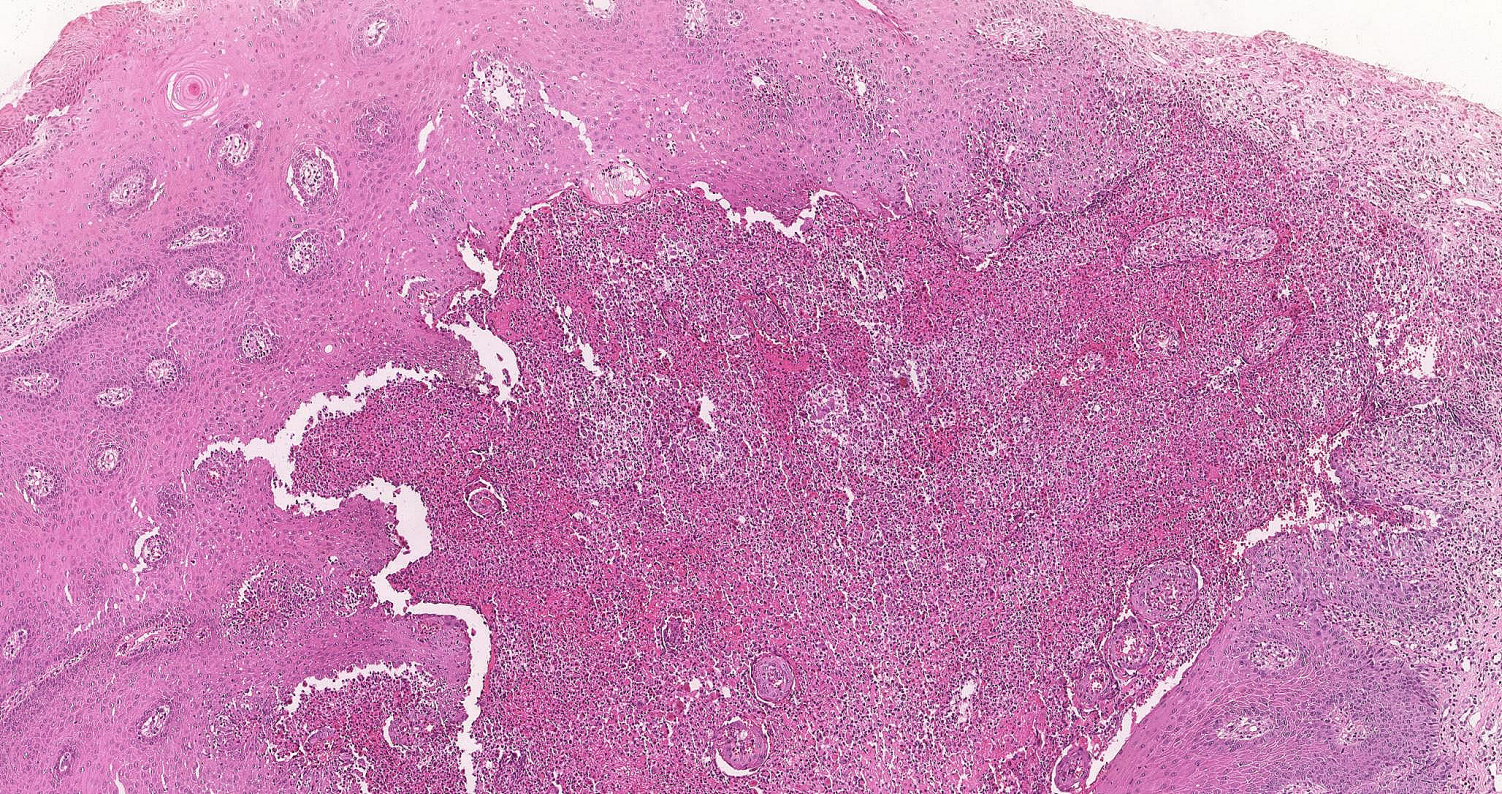
Histopathological photomicrograph showing intraepithelial and subepithelial pustules containing neutrophils and eosinophils. H&E, x10

**Fig. 6:**
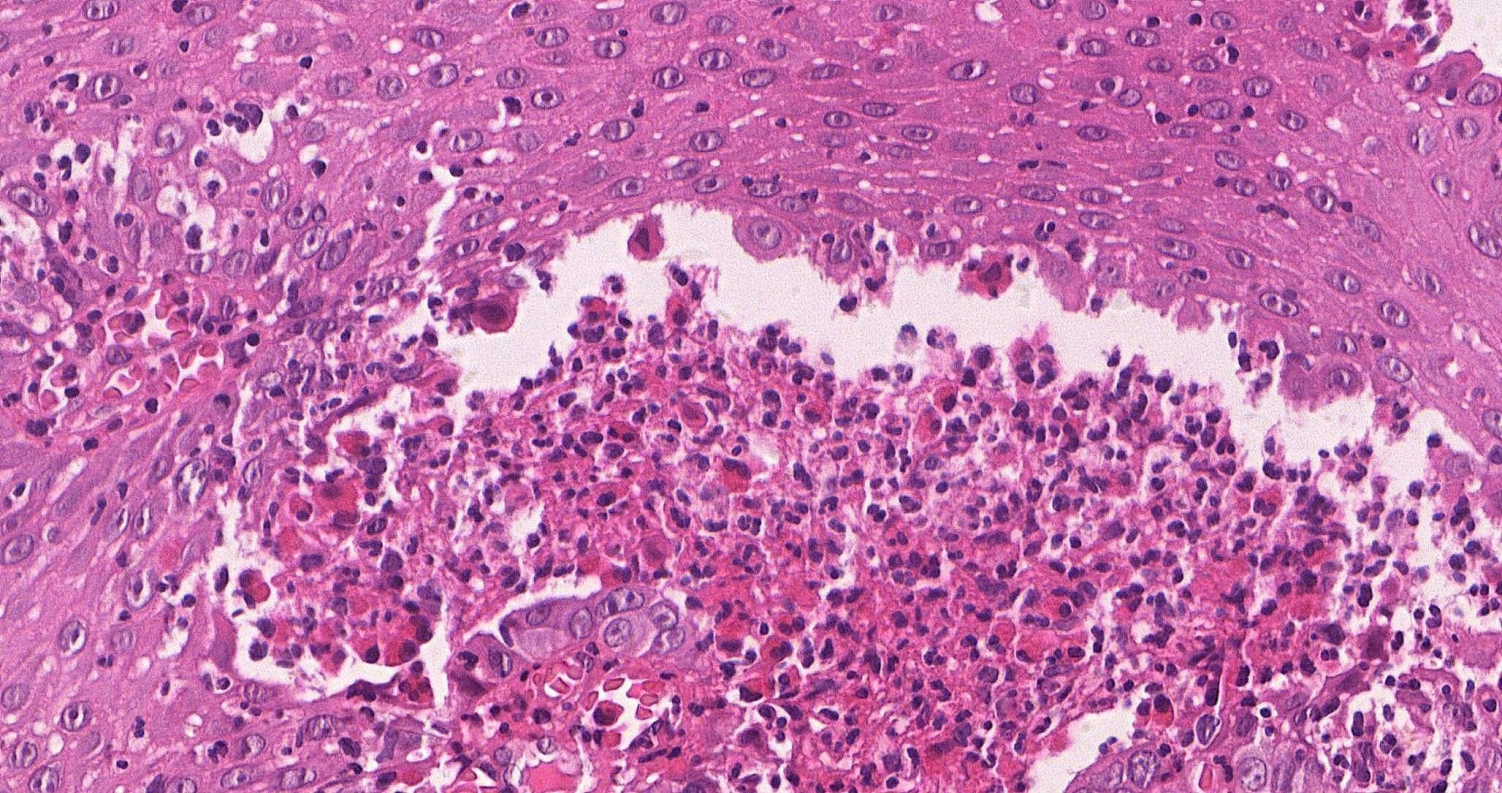
Histopathological photomicrograph showing acantholysis with intraepithelial clefs formation. H&E, x70

The microscopic exam showed pseudoepitheliomatous hyperplasia with widespread eosinophilic and neutrophilic infiltration, acantholysis with intraepithelial clefs formation. Intraepithelial and subepithelial pustules containing neutrophils and eosinophils were observed (Figs. 5 and 6).

Although no symptom related to inflammatory bowel disease was reported, presence of oral lesions prompted referral to a gastroenterologist and colonoscopy confirmed diagnosis of ulcerative colitis. Infliximab and systemic corticosteroids (40 mg of prednisone/day, which gradually reduced in dose) were prescribed and both lesions and symptoms disappeared after 7 days of treatment. The patient is being followed by a gastroenterologist.

## Materials and methods

This study followed the PRISMA Statement guidelines [8] and is registered in PROSPERO (CRD42022352051).

### Search strategies

An electronic search without time restrictions for publications published in either English, Portuguese or Spanish was undertaken in October 2021, with a complementary search conducted on August 1st 2022, in the following databases: PubMed/Medline, Web of Science, and Science Direct. Terms that were used in the past but no longer are in use were also used, to minimize risk of missing early reports of this condition in the search. The following terms were used in the search strategies:

(“Pyostomatitis vegetans”) OR (“pyodermatitis vegetans”) OR (“pyodermite végétante”) OR (“dermatitis vegetans”) OR (“pyoderma vegetans”) OR (“pyodermatitis-pyostomatitis vegetans”) OR (“pyostomatitis-pyodermatitis vegetans”) OR (“oro-facial granulomatosis”).

Google Scholar was checked. The reference list of identified studies and elevant reviews on the subject were also examined to identify possible additional studies. In addition, publications with lesions identified by other authors as being pyostomatitis vegetans, even if the term “pyostomatitis vegetans” was not included in the title of the article, were re-evaluated by two of the authorsin the present study.

### Definitions

Inflammatory bowel disease (IBD) consists of a group of autoimmune diseases characterized by inflammation of the intestines. The inflammatory condition is presented in two major forms, namely ulcerative colitis and Crohn’s disease. The symptoms commonly presented by patients with IBD include abdominal symptoms, including diarrhea, pain, bloody stools, and vomiting [9].

### Inclusion and exclusion criteria

Publications reporting cases of PSV in which diagnosis confirmed by the pathological examination of oral or skin lesions, (with characteristic intra-epithelial and/or subepithelial micro abscesses containing eosinophils), and presence of inflammatory bowel disease (IBD) were eligible for our analysis. This included clinical trials, cohort studies, case-control studies, cross-sectional studies, case series, and case reports were included.

Cases of injuries that affected only the perioral skin were excluded. Exclusion criteria also consisted of immunohistochemical studies, histomorphometric studies, radiological studies, genetic expression studies, histopathological studies, cytological studies, cell proliferation/apoptosis studies, in vitro studies, and review papers, unless any of these publication categories had reported any cases fulfilling the aforementioned inclusion criteria.

### Study selection

The titles and abstracts of all reports identified through the electronic searches were read independently by the authors. For studies appearing to meet the inclusion criteria, or for which there were insufficient data in the title and abstract to make a clear decision, the full report was obtained. Disagreements were resolved by discussion between the authors. The pathological reports of the lesions were thoroughly assessed by two of the authors (R.S.G. and R.R.M.C.) of the present study in order to confirm the diagnosis of PSV.

RefWorks Reference Management Software (Ex Libris, Jerusalem, Israel) was used in order to detect duplicate references in different electronic databases.

### Data extraction

The review authors independently extracted data using specially designed data extraction forms. Any disagreements were resolved by discussion. For each of the identified studies included, the following data were then extracted on a standard form, when available: patient’s sex and, location of the oral lesions, presence of skin lesions, clinical symptoms (for the oral lesions), presence of “snail track” appearance of the oral lesions, microscopic presence of intraepithelial clefting/splitting in the oral lesions, presence of lymphadenopathy, leukocytosis and/or eosinophilia on bloodwork, associated IBD clinical symptoms, timing of IBD diagnosis in relation to detection of oral lesions (before, concomitantly, or after the appearance of the oral lesions), results of oral and/skin lesions immunofluorescence analysis, time between the first signs of oral lesions to the beginning of a treatment, treatment adopted, remission, recurrence, and follow-up period.

### Analyses

Descriptive analysis was performed based on mean, standard deviation (SD), and percentage values. Data was analyzed using IBM SPSS Statistics for Windows, version 28 (IBM Corp., Armonk, NY, USA).

## Results

### Literature search

The study selection process is summarized in Figure S1 (see Supplementary Material). The search strategy resulted in 1600 entries (270 in Pubmed, 889 in Web of Science, 441 in ScienceDirect). Search in Google Scholar resulted in 16 eligible papers not found in the three main databases. A total of 448 articles were duplicates. Reviewers independently screened the abstracts for those articles related to the aim of the review. This yielded 1168 studies of which 1057 were excluded for not being related to the topic or not presenting clinical cases. Five additional cases were identified through hand-searching of journals and reviewing the reference lists of selected studies. The full-text reports of the remaining 116 articles led to the exclusion of 54 because they did not meet the inclusion criteria. Thus, a total of 62 publications were included in the review (list of references in the Supplementary Material). We also added our own case to the final list.

### Description of the studies and analyses

There were 77 cases of PSV in patients with oral lesions and an associated IBD, including the present case. The reported IBD were ulcerative colitis (*n* = 50), Crohn’s disease (*n* = 24), non-specified colitis (*n* = 2), and post-infective irritable bowel syndrome (*n* = 1).

Table 1 summarizes the demographic and clinical features of the cases of PSV identified.

**Table 1:** Demographic and clinical features of the cases of pyostomatitis vegetans.Information on the variables is not always available for all cases is not always available for all cases

Factors	Cases
N	77
	n (%)
Sex	
Male/Female	36 (46.8)/41 (53.2)
Pain ^a^	
Yes/No	30 (60.0)/20 (40.0)
Snail track appearance ^a^	
Yes/No	73 (94.8)/4 (5.2)
Intraepithelial clefting ^a^	
Yes/No	51 (81.0)/12 (19.0)
Skin lesions	
Yes/No	40 (55.6)/32 (44.4)
Lymphadenopathy	
Yes/No	5 (25.0)/15 (75.0)
IBD diagnosis	
Before/Concomitantly/After oral lesions	45 (60.8)/17 (23.0)/12 (16.2)
Gastrointestinal symptoms	
Yes/No	48 (76.2)/15 (23.8)
Leukocytosis	
Yes/No	6 (20.0)/24 (80.0)
Eosinophilia	
Yes/No	30 (63.8)/17 (36.2)
Oral biopsy ^b^	
Yes/No	68 (88.3)/9 (11.7)
Positive immunofluorescence ^c^	
Yes/No	17 (37.8)/28 (62.2)
IgA only	2
IgG only	9
IgA and IgG	6
Location of oral lesions	
Labial mucosa	
Yes/No	44 (57.9)/32 (42.1)
Buccal mucosa	
Yes/No	47 (61.8)/29 (38.2)
Lips vermilion	
Yes/No	25 (32.5)/52 (67.5)
Gingiva	
Yes/No	45 (59.2)/31 (40.8)
Palate	
Yes/No	27 (36.8)/48 (63.2)
Tongue	
Yes/No	19 (26.3)/56 (73.7)
Mouth floor	
Yes/No	9 (13.2)/66 (86.8)
	Mean ± SD (min - max)
Age at clinical consultation (years)	37.0 ± 16.0 (9–76) (*n* = 77)
Duration of oral lesions previously to treatment (months)	6.6 ± 12.5 (0.3–72) (*n* = 47)
Time between IBD diagnosis and oral lesions ^d^ (months)	102.5 ± 69 (12–288) (*n* = 33)
Follow-up ^a^ (months)	26.0 ± 48.2 (0.3–228) (*n* = 56)

IBD: inflammatory bowel disease.

IgA: immunoglobulin A.

IgG: immunoglobulin G.

SD: standard deviation.

^a^Valid for oral lesions only.

^b^Cases in which no oral biopsy was performed had at least biopsy of a skin lesion.

^c^Of either oral or skin biopsy samples, or both.

^d^For the cases in which the diagnosis of an IBD was confirmed before the occurrence of oral lesions.

Forty patients had concomitant skin lesions in the following areas and the same patient could have more than one region affected: scalp (*n* = 23), trunk/chest (*n* = 13), leg/thigh (*n* = 12), eyes/eyelids (*n* = 11), groin (*n* = 10), arms (*n* = 9), axilla (*n* = 8), neck (*n* = 8), genitals (*n* = 7), face (*n* = 6), abdomen (*n* = 6), back/flanks (*n* = 4), anus/perianal (*n* = 3), buttocks (*n* = 2), pubic region (*n* = 2), ear (*n* = 2), feet (*n* = 2), and nose/nasal mucosa (*n* = 2).

Immunofluorescence analysis of oral specimens was usually positive for immunoglobulins IgG, IgA, and C3, but only in two cases tissue analysis was simultaneously positive for al three factors.

Management of PSV typically involves topical and/or systemic corticosteroids. Systemic corticosteroids and/or immunosuppressives are usually prescribed for the associated IBD. There was no clear consensus regarding the therapeutic protocol in the literature. The main systemic drugs were corticosteroids (prednisolone, beclomethasone), antirheumatic drugs (sulfasalazine, mesalazine), monoclonal antibody (infliximab, adalimumab) immunosuppressives (azathioprine, methotrexate), antibiotics (dapsone), or a combination of these. The most common drugs used topically or as a mouth rinse included betamethasone, dexamethasone, clobetasol propionate, triamcinolone acetonide, mometasone furoate, hydrocortisone. When information was available, and irrespective of the drug used, oral lesions recurred in 26 (53.1%) out of 49 cases rafter drug cessation or dose reduction.

## Discussion

Our review shows that cases of PSV are slightly more prevalent in males and that oral lesions most commonly affect buccal and labial mucosa, and gingiva. Soft and hard palate, lips vermilion, and tongue are less affected and lesions involving floor of the mouth are least frequent. The most common clinical presentation for oral lesions is Snail track appearance with nearly 95% of the cases presenting this feature. In addition, 60% of oral lesions are symptomatic especially when eating. Moreover, patients may concomitantly present with skin lesions, virtually in any part of the body, in about half of the cases.

As seen in Table 1, diagnosis of IBD was confirmed before the diagnosis of PSV in about 60% of the patients. It is important to realize that lack of gastrointestinal symptoms does not rule out IBD. In fact, 23.8% of the patients did not present any gastrointestinal symptom despite confirmed diagnosis of IBD. This finding supports the importance of evaluating patients for IBD even if patients with PSV have never presented gastrointestinal symptoms.

Diagnosis of PSV relies on sampling of oral lesions for histopathological analysis. Although presence of intraepithelial clefts containing eosinophils are typical microscopic feature which was reported in 81% of cases in our review. In some cases, subepithelial micro abscesses containing eosinophils supported PSV diagnosis.

Information regarding the number of circulating eosinophils was available in 47 cases, and in 30 (63.8%) eosinophilia was detected. This finding may support utility of complete hemogram as an auxiliary test when diagnosis of PSV is suspected.

The differential diagnosis of PSV includes a variety of conditions the most challenging of which in terms of differentiation is pemphigus vegetans. Pemphigus vegetans is a rare variant of pemphigus vulgaris characterized by vegetating plaques in the flexures [10]. Although skin is the primary site of involvement in pemphigus vegetans, oral lesions are occasionally reported [10] and could mimic PSV [11, 12]. In pemphigus vegetans, direct immunofluorescence generally shows intercellular deposition of immunoglobulin G (IgG) [13]. Among the 45 cases of PSV we studied, 15 (33.3%) were also positive for IgG. This finding may suggest underlying associations between autoantibodies and PSV [3]. In fact, some studies suggest that PSV may be a part of a spectrum of diseases, including pemphigus vegetans [12]. Since delivery and pregnancy are systemic conditions possibly related to pemphigus vegetans [14], we also considered this hypothesis. Although we did not perform direct immunofluorescence at the time of tissue biopsy, diagnosis of ulcerative colitis at colonoscopy favored diagnosis of PSV.

There was no clear established drug therapy protocol for PSV in the literature. Management of PSV and associated IBD primarily involved of topical and/or systemic corticosteroids, monoclonal antibody, immunosuppressives, antibiotics, or a combination of these. It is difficult to determine which drug would provide a better efficacy against PSV, due to the plethora of drugs used in the cases described in the literature. This is compounded by differences in dosing and duration of therapy as well as possible addictive effect of systemic medications used concomitantly to manage the underling IBD. Nevertheless, our analysis showed increased risk of recurrence for PSV when the medication dose is reduced or fully interrupted. This was observed in more than half of the cases when follow-up information was available.

The results of our study must be interpreted with caution because it has several limitations. First, most of included publications were single case reports. This is understandable in light of rare occurrence/reporting of a disease with oral manifestations. In addition, information about all variables were not available across all studies and could have affected the quality of the statistical analyses [15]. Moreover, extensive variation of therapies reported in the cases reviewed made it very difficult, not to say impossible, to establish a clear therapeutic protocol for achieving remission of PSV.

## Conclusions

The absence of cutaneous lesions does not exclude PSV diagnosis. The percentage of IBD diagnosed concomitantly or following oral involvement highlights the value of gastrointestinal investigation in patients with erosive vesiculopustular oral lesions, even in absence of abdominal symptoms. Features that are helpful in establishing the diagnosis of PSV are snail track appearance, an associated IBD (which not always is symptomatic), microscopic intraepithelial clefting of oral lesions, and peripheral blood eosinophilia. A gold standard for therapeutic management of PSV does not exist and high-level evidence is limited.

### Electronic supplementary material

Below is the link to the electronic supplementary material.

Supplementary Material 1

## Data Availability

All data generated or analyzed during this study are included in this published article. Data for the systematic review were collected from the included studies, which list is found in the supplementary information files.
