# Supplementary material for: Pyodermatitis-pyostomatitis vegetans: a case report and systematic review focusing on oral involvement
Source: Oral Maxillofac Surg. 2024 Mar 12;28(3):1405–14. doi: 10.1007/s10006-024-01234-1 (PMC11330387; doi:10.1007/s10006-024-01234-1)
Supplement: Supplementary file 1 — Supplementary Material 1 [file 10006_2024_1234_MOESM1_ESM.docx]

**SUPPLEMENTARY MATERIAL**

Figure S1. Study screening process.


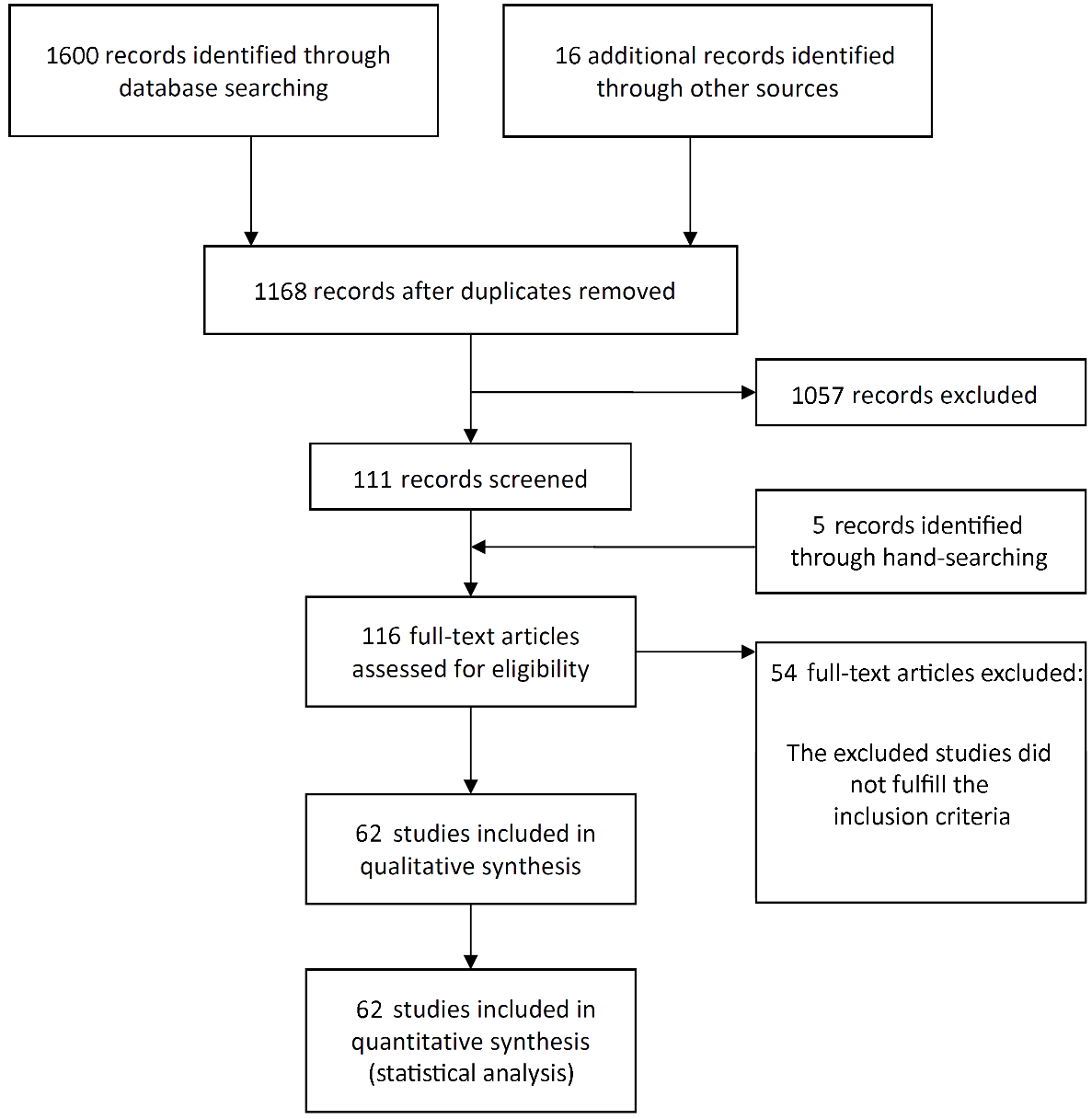


Publications included in the review

1. Abellaneda C, Mascaró JM Jr, Vázquez MG, Pablo IM, Iranzo P. All that glitters is not pemphigus: Pyodermatitis-pyostomatitis vegetans misdiagnosed as IgA pemphigus for 8 years. Am J Dermatopathol. 2011 Feb;33(1):e1-6.
2. Ahn BK, Kim SC. Pyodermatitis-pyostomatitis vegetans with circulating autoantibodies to bullous pemphigoid antigen 230. J Am Acad Dermatol. 2004 May;50(5):785-8.
3. Atarbashi-Moghadam S, Lotfi A, Atarbashi-Moghadam F. Pyostomatitis Vegetans: A Clue for Diagnosis of Silent Crohn's Disease. J Clin Diagn Res. 2016 Dec;10(12):ZD12-ZD13.
4. Ayangco L, Rogers RS 3rd, Sheridan PJ. Pyostomatitis vegetans as an early sign of reactivation of Crohn's disease: a case report. J Periodontol. 2002 Dec;73(12):1512-6.
5. Ballo FS, Camisa C, Allen CM. Pyostomatitis vegetans. Report of a case and review of the literature. J Am Acad Dermatol. 1989 Aug;21(2 Pt 2):381-7.
6. Bardasi G, Romagnoli A, Foschini MP, Mantovani A, Alvisi P. Pyostomatitis vegetans in a pediatric patient with ulcerative colitis: case report of a rare pediatric inflammatory bowel disease extraintestinal manifestation and review of the literature. Eur J Gastroenterol Hepatol. 2020 Jul;32(7):889-892.
7. Bens G, Laharie D, Beylot-Barry M, Vergier B, Noblesse I, Beylot C, Amouretti M. Successful treatment with infliximab and methotrexate of pyostomatitis vegetans associated with Crohn's disease. Br J Dermatol. 2003 Jul;149(1):181-4.
8. Bertlich I, Gauss A, Schäkel K, Enk A, Hoffmann JHO. Pyodermatitis-pyostomatitis vegetans with histological and immunohistological aspects of autoimmune blistering disease treated with infliximab. J Dtsch Dermatol Ges. 2019 May;17(5):540-542.
9. Berzin D, Lahad A, Weiss B, Barzilai A, Greenberger S. Inflammatory bowel disease presenting with pyodermatitis-pyostomatitis vegetans in a pediatric patient: A case report and review of the literature. Pediatr Dermatol. 2021 Jul;38(4):868-871.
10. Calobrisi SD, Mutasim DF, McDonald JS. Pyostomatitis vegetans associated with ulcerative colitis. Temporary clearance with fluocinonide gel and complete remission after colectomy. Oral Surg Oral Med Oral Pathol Oral Radiol Endod. 1995 Apr;79(4):452-4.
11. Cataldo E, Covino MC, Tesone PE. Pyostomatitis vegetans. Oral Surg Oral Med Oral Pathol. 1981 Aug;52(2):172-7.
12. Chan SW, Scully C, Prime SS, Eveson J. Pyostomatitis vegetans: oral manifestation of ulcerative colitis. Oral Surg Oral Med Oral Pathol. 1991 Dec;72(6):689-92.
13. Chaudhry SI, Philpot NS, Odell EW, Challacombe SJ, Shirlaw PJ. Pyostomatitis vegetans associated with asymptomatic ulcerative colitis: a case report. Oral Surg Oral Med Oral Pathol Oral Radiol Endod. 1999 Mar;87(3):327-30.
14. Clark LG, Tolkachjov SN, Bridges AG, Camilleri MJ. Pyostomatitis vegetans (PSV)-pyodermatitis vegetans (PDV): A clinicopathologic study of 7 cases at a tertiary referral center. J Am Acad Dermatol. 2016 Sep;75(3):578-584.
15. De Giorgi V, Trane L, Silvestri F, Venturi F, Zuccaro B, Scarfì F. Pyodermatitis-Pyostomatitis Vegetans in Active Inflammatory Bowel Disease. Am J Gastroenterol. 2021 May 31.
16. Dimmock M, Mendes LC, Albluwi S, Paul C, Thomas C, Laurencin S, Cousty S. An oral manifestation of IBD: Pyostomatitis vegetant, about two cases. J Stomatol Oral Maxillofac Surg. 2019 Sep;120(4):375-377.
17. Dodd EM, Howard JR, Dulaney ED, Rosenthal SI, Wanna MR, Farah RS. Pyodermatitis-pyostomatitis vegetans associated with asymptomatic inflammatory bowel disease. Int J Dermatol. 2017 Dec;56(12):1457-1459.
18. Dupuis EC, Haber RM, Robertson LH. Pyoblepharitis Vegetans in Association With Pyodermatitis-Pyostomatitis Vegetans: Expanding the Spectrum of a Rare, Multisystem Disorder. J Cutan Med Surg. 2016 Mar-Apr;20(2):163-5.
19. Ficarra G, Baroni G, Massi D. Pyostomatitis vegetans: cellular immune profile and expression of IL-6, IL-8 and TNF-alpha. Head Neck Pathol. 2010 Mar;4(1):1-9.
20. Ficarra G, Cicchi P, Amorosi A, Piluso S. Oral Crohn's disease and pyostomatitis vegetans. An unusual association. Oral Surg Oral Med Oral Pathol. 1993 Feb;75(2):220-4.
21. Forman L. Two cases of pyodermite vegetante (hallopeau): an eosinophilic pustular and vegetating dermatitis with conjunctival, oral and colonic involvement. Proc R Soc Med. 1965 Apr;58(4):244-9.
22. Gara S, Souissi A, Mokni M. Pyodermatitis Pyostomatitis Vegetans. JAMA Dermatol. 2020 Mar 1;156(3):335.
23. Gheisari M, Zerehpoosh FB, Zaresharifi S. Pyodermatitis-pyostomatitis vegetans: a case report and review of literature. Dermatol Online J. 2020 May 15;26(5):13030/qt5871q750.
24. Hansen LS, Silverman S Jr, Daniels TE. The differential diagnosis of pyostomatitis vegetans and its relation to bowel disease. Oral Surg Oral Med Oral Pathol. 1983 Apr;55(4):363-73.
25. Healy CM, Farthing PM, Williams DM, Thornhill MH. Pyostomatitis vegetans and associated systemic disease. A review and two case reports. Oral Surg Oral Med Oral Pathol. 1994 Sep;78(3):323-8.
26. Hou PC, Huang HY, Lee JYY, Hsu CK. Pyostomatitis vegetans following coronavirus disease 2019 vaccination in a patient with ulcerative colitis. Journal of Dermatology. 2022. (in press)
27. Kalman RS, Gjede JM, Farraye FA. Pyostomatitis vegetans in a patient with fistulizing Crohn's disease. Clin Gastroenterol Hepatol. 2013 Dec;11(12):A24.
28. Kamal N, Bookwalter A, Foss R, Cross RK. Pyostomatitis Vegetans: An Unusual Oral Manifestation of Inflammatory Bowel Disease. Am J Gastroenterol. 2020 Sep;115(9):1385.
29. Kim TH, Kim SC. Pyodermatitis-Pyostomatitis Vegetans Associated with Crohn's Disease. Ann Dermatol. 2015 Oct;27(5):624-5.
30. Kitayama A, Misago N, Okawa T, Iwakiri R, Narisawa Y. Pyodermatitis-pyostomatitis vegetans after subtotal colectomy for ulcerative colitis. J Dermatol. 2010 Aug;37(8):714-7.
31. Konstantopoulou M, O'Dwyer EM, Steele JC, Field EA, Lewis MA, Macfarlane AW. Pyodermatitis-pyostomatitis vegetans complicated by methicillin-resistant Staphylococcus aureus infection. Clin Exp Dermatol. 2005 Nov;30(6):666-8.
32. Leibovitch I, Ooi C, Huilgol SC, Reid C, James CL, Selva D. Pyodermatitis-pyostomatitis vegetans of the eyelids case report and review of the literature. Ophthalmology. 2005 Oct;112(10):1809-13.
33. Lewis JE, Beutner EH. Pseudo-pyostomatitis vegetans. Int J Dermatol. 1995 Sep;34(9):656-7.
34. Lopez-Jornet P, Gomez-Garcia F, Camacho-Alonso F. Pyostomatitis vegetans. Clinical marker of ulcerative colitis. N Y State Dent J. 2012 Mar;78(2):36-7.
35. Lourenço SV, Hussein TP, Bologna SB, Sipahi AM, Nico MM. Oral manifestations of inflammatory bowel disease: a review based on the observation of six cases. J Eur Acad Dermatol Venereol. 2010 Feb;24(2):204-7.
36. Markiewicz M, Suresh L, Margarone J 3rd, Aguirre A, Brass C. Pyostomatitis vegetans: A clinical marker of silent ulcerative colitis. J Oral Maxillofac Surg. 2007 Feb;65(2):346-8.
37. McCarthy FP. Pyostomatitis vegetans. Archives of Dermatology and Syphilology; 1949 Nov 1;60(5_PART_I):750.
38. Merkourea SS, Tosios KI, Merkoureas S, Sklavounou-Andrikopoulou A. Pyostomatitis vegetans leading to Crohn's disease diagnosis. Ann Gastroenterol. 2013;26(2):187.
39. Mijandrusić-Sincić B, Licul V, Gorup L, Brncić N, Glazar I, Lucin K. Pyostomatitis vegetans associated with inflammatory bowel disease--report of two cases. Coll Antropol. 2010 Apr;34 Suppl 2:279-82.
40. Mizukami Y, Imanishi H, Tateishi C, Kaneshiro S, Sowa-Osako J, Ohsawa M, Tsuruta D. Successful treatment of pyostomatitis vegetans with ulcerative colitis using dapsone without systemic steroids. J Dermatol. 2019 Sep;46(9):e316-e317.
41. Molnár T, Farkas K, Nagy F, Vass N, Szepes Z, Tiszlavicz L, Wittmann T. Third case: Another pediatric patient with pyostomatitis vegetans and oral granuloma as one of the initial symptoms of Crohn's disease. Inflamm Bowel Dis. 2011 Sep;17(9):E122-3.
42. Naish JM, Batchvarov BD, Lawoyin VL. A case of ulcerative colitis and pyostomatitis vegetans in an African. Gut. 1970 Jan;11(1):38-40.
43. Nanda A, Lazarevic V, Rajy JM, Almasry IM, AlSabah H, AlLafi A. Spectrum of autoimmune bullous diseases among children in Kuwait. Pediatr Dermatol. 2020 Oct 11.
44. Neville BW, Smith SE, Maize JC, Laden SA, Denton WT. Pyostomatitis vegetans. Am J Dermatopathol. 1985 Feb;7(1):69-77.
45. Nico MM, Hussein TP, Aoki V, Lourenço SV. Pyostomatitis vegetans and its relation to inflammatory bowel disease, pyoderma gangrenosum, pyodermatitis vegetans, and pemphigus. J Oral Pathol Med. 2012 Sep;41(8):584-8.
46. Pazheri F, Alkhouri N, Radhakrishnan K. Pyostomatitis vegetans as an oral manifestation of Crohn's disease in a pediatric patient. Inflamm Bowel Dis. 2010 Dec;16(12):2007.
47. Petruzzi M, Della Vella F. Pyostomatitis Vegetans. N Engl J Med. 2021 Nov 25;385(22):e77.
48. Ruiz-Roca JA, Berini-Aytés L, Gay-Escoda C. Pyostomatitis vegetans. Report of two cases and review of the literature. Oral Surg Oral Med Oral Pathol Oral Radiol Endod. 2005 Apr;99(4):447-54.
49. Shah S, Cotliar J. Images in clinical medicine. Pyostomatitis vegetans. N Engl J Med. 2013 May 16;368(20):1918.
50. Sollecito T, Stoopler E, Rangarajan S, Pinto A. Pyostomatitis Vegetans And Orofacial Granulomatosis: A Case Report And Review Of The Literature. The Internet Journal of Anesthesiology. Internet Scientific Publishers, LLC; 2003;14(2).
51. Soriano ML, Martínez N, Grilli R, Fariña MC, Martín L, Requena L. Pyodermatitis-pyostomatitis vegetans: report of a case and review of the literature. Oral Surg Oral Med Oral Pathol Oral Radiol Endod. 1999 Mar;87(3):322-6.
52. Steele L, Jeetle S, Kok K, Cunningham M, Goldsmith P. Pyodermatitis vegetans-pyostomatitis vegetans with ocular involvement. Clin Exp Dermatol. 2020 Sep 25.
53. Stingeni L, Tramontana M, Bassotti G, Bianchi L, Lisi P. Pyodermatitis-pyostomatitis vegetans and antibullous pemphigoid antigen 180 autoantibodies: a casual association? Br J Dermatol. 2015 Mar;172(3):811-3.
54. Storwick GS, Prihoda MB, Fulton RJ, Wood WS. Pyodermatitis-pyostomatitis vegetans: a specific marker for inflammatory bowel disease. J Am Acad Dermatol. 1994 Aug;31(2 Pt 2):336-41.
55. Thornhill MH, Zakrzewska JM, Gilkes JJ. Pyostomatitis vegetans: report of three cases and review of the literature. J Oral Pathol Med. 1992 Mar;21(3):128-33.
56. VanHale HM, Rogers RS 3rd, Zone JJ, Greipp PR. Pyostomatitis vegetans. A reactive mucosal marker for inflammatory disease of the gut. Arch Dermatol. 1985 Jan;121(1):94-8.
57. Wang H, Qiao S, Zhang X, Liu C. A case of pyodermatitis-pyostomatitis vegetans. Am J Med Sci. 2013 Feb;345(2):168-71.
58. Werchniak AE, Storm CA, Plunkett RW, Beutner EH, Dinulos JG. Treatment of pyostomatitis vegetans with topical tacrolimus. J Am Acad Dermatol. 2005 Apr;52(4):722-3.
59. Wray D. Pyostomatitis vegetans. Br Dent J. 1984 Nov 10;157(9):316-8.
60. Wu YH, Chang JY, Chen HM, Wang YP. Pyostomatitis vegetans: An oral manifestation of inflammatory bowel disease. J Formos Med Assoc. 2015 Jul;114(7):672-3.
61. Xiamei Z, Hong H, Xiaosheng H. Rare Mucocutaneous Manifestations of Ulcerative Colitis: A Case report of Pyostomatitis Vegetans and Sweet's Syndrome. Oral Surgery, Oral Medicine, Oral Pathology and Oral Radiology. 2022 (in press).
62. Zegarelli EV, Kutscher AH. Oral "pyoderma". Report of two cases. Am J Dig Dis. 1962 Mar;7:281-8.
